# (Acetyl­acetonato-κ^2^ *O*,*O*′)carbon­yl[tris­(4-chloro­phen­yl)phosphane-κ*P*]rhodium(I)

**DOI:** 10.1107/S1600536812012536

**Published:** 2012-03-31

**Authors:** Nathan C. Antonels, Reinout Meijboom

**Affiliations:** a Research Center for Synthesis and Catalysis, Department of Chemistry, University of Johannesburg (APK Campus), PO Box 524, Auckland Park, Johannesburg 2006, South Africa

## Abstract

The title compound, [Rh(C_5_H_7_O_2_)(C_18_H_12_Cl_3_P)(CO)], contains the bidentate acetyl­acetonate ligand coordinated to the Rh^I^ atom, forming a chelate ring [Rh—O = 2.0327 (15) and 2.0613 (14) Å]. The Rh^I^ atom is additionally coordinated by one P [Rh—P = 2.2281 (6) Å] and one carbonyl C [Rh—C = 1.812 (2) Å] atom, resulting in a slightly distorted square-planar geometry. The mol­ecules are packed to minimize steric hindrance with the phosphanes positioned above and below the slightly distorted square geometrical plane.

## Related literature

For background literature on the catalytic activity of rhodium–phosphane compounds, see: Carraz *et al.* (2000 ▶); Moloy & Wegman (1989 ▶); Bonati & Wilkinson (1964 ▶). For related rhodium compounds, see: Brink *et al.* (2007 ▶); Erasmus & Conradie (2011 ▶); Leipoldt *et al.* (1978 ▶); Steynberg *et al.* (1987 ▶).
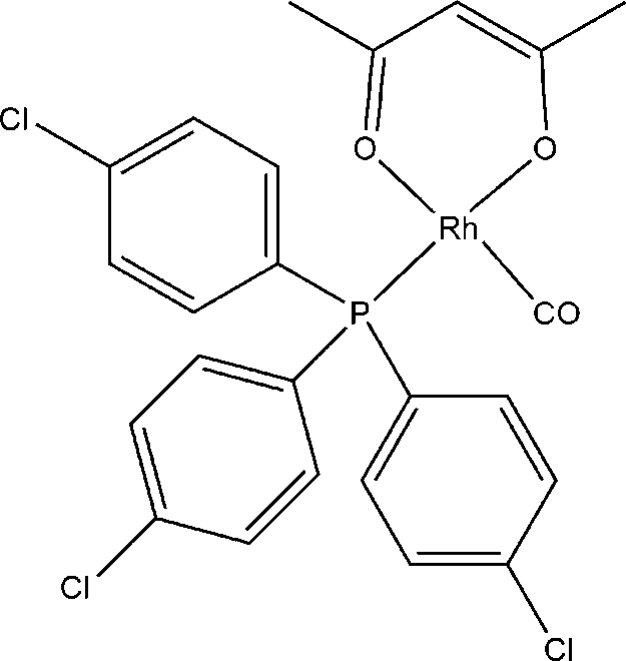


## Experimental

### 

#### Crystal data

[Rh(C_5_H_7_O_2_)(C_18_H_12_Cl_3_P)(CO)]
*M* *_r_* = 595.62
Triclinic, 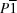

*a* = 9.6528 (17) Å
*b* = 11.535 (2) Å
*c* = 12.875 (2) Å
α = 65.211 (3)°
β = 72.095 (4)°
γ = 72.757 (4)°
*V* = 1214.9 (4) Å^3^
*Z* = 2
Mo *K*α radiation
μ = 1.12 mm^−1^
*T* = 100 K
0.18 × 0.13 × 0.12 mm

#### Data collection

Bruker APEX DUO 4K CCD diffractometer
Absorption correction: multi-scan (*SADABS*; Bruker, 2008 ▶) *T*_min_ = 0.826, *T*_max_ = 0.877
19382 measured reflections
6083 independent reflections
5395 reflections with *I* > 2σ(*I*)
*R*_int_ = 0.029

#### Refinement

*R*[*F* ^2^ > 2σ(*F* ^2^)] = 0.027
*wR*(*F* ^2^) = 0.063
*S* = 1.01
6083 reflections
291 parameters
H-atom parameters constrained
Δρ_max_ = 0.50 e Å^−3^
Δρ_min_ = −0.48 e Å^−3^

### 

Data collection: *APEX2* (Bruker, 2010 ▶); cell refinement: *SAINT* (Bruker, 2008 ▶); data reduction: *SAINT*; program(s) used to solve structure: *SIR97* (Altomare *et al.*, 1999 ▶); program(s) used to refine structure: *SHELXL97* (Sheldrick, 2008 ▶); molecular graphics: *DIAMOND* (Brandenburg & Putz, 2005 ▶); software used to prepare material for publication: *publCIF* (Westrip, 2010 ▶) and *WinGX* (Farrugia, 1999 ▶).

## Supplementary Material

Crystal structure: contains datablock(s) global, I. DOI: 10.1107/S1600536812012536/fk2054sup1.cif

Structure factors: contains datablock(s) I. DOI: 10.1107/S1600536812012536/fk2054Isup2.hkl

Additional supplementary materials:  crystallographic information; 3D view; checkCIF report

## Figures and Tables

**Fig. 1.**
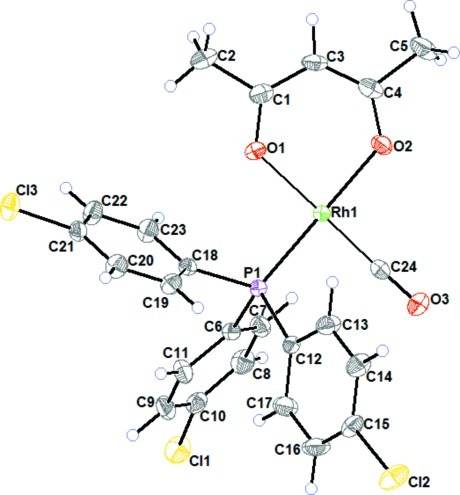
Molecular structure of the title compound. Displacement ellipsoids are drawn at the 30% probability level.

## supplementary crystallographic information

### Comment

Acetylacetonate has two O-donor atoms with equivalent σ-electron donor capabilities. The high symmetry of dicarbonyl(acetylacetonate)rhodium(I) complexes promotes easy carbonyl displacement of either carbonyl group with a variety of phosphanes, phosphites and arsines (Bonati and Wilkinson, 1964). These dicarbonyl(acetylacetonate)rhodium(I) compounds are typically used in methyl iodide oxidative addition studies (Erasmus and Conradie, 2011). This study is part of ongoing research into induced effects of various phosphanes coordinated to Rh(I) centers. Previous work illustrating the catalytic importance of the rhodium(I) square-planar moieties has been conducted on rhodium mono- and di-phosphane complexes containing the symmetrical bidentate ligand, acac (acac = acetylacetonate) (Moloy and Wegman, 1989). Symmetrical di-phosphane ligands result in the production of acetaldehyde, whereas unsymmetrical di-phosphane ligands are more stable and efficient catalysts for the carbonylation of methanol to acetic acid (Carraz *et al.*, 2000). In the title compound, [Rh(acac)(CO){P(4—Cl—C_6_H_4)_3}] (acac = acetylacetonate), the coordination around the Rh atom shows a slightly distorted square-planar arrangement, illustrated by C24—Rh1—P1 and O1—Rh1—O2 angles of 88.08 (7)° and 89.37 (6)°, respectively and a distance of 0.0015 (2) Å for Rh1 from the mean coordination plane. The Rh—C and Rh—P bond lengths are 1.812 (2) Å and 2.2280 (6) Å respectively. A larger *trans* influence of the phosphane ligand with respect to the carbonyl ligand is indicated by the longer Rh—O2 (2.0613 (14) Å) bond compared to Rh—O1 (2.0327 (15) Å) bond which is *trans* to the carbonyl ligand. The molecular geometries are similar to those observed from the closely related compounds known from Steynberg *et al.*(1987) and Leipoldt *et al.*(1978). These compounds show similar spectroscopic properties to those as discussed by Brink *et al.*(2007).

### Experimental

A solution of [PCy_2_(4-Me_2_NC_6_H_4_)] (63.6 mg, 0.174 mmol) in acetone (3 cm^3^) was slowly added to a solution of [Rh(acac)(CO)_2_] (44.5 mg, 0.172 mmol) in acetone (4 cm^3^). The solvent was removed and recrystallized from dichloromethane. Slow evaporation of the solvent afforded the title compound as yellow crystals (yield: 85.5 mg, 83%). Spectroscopic data: ^31P^{H} NMR (CDCl_3_, 161.98 MHz, p.p.m.): 47.55 p.p.m. [^1^J(Rh—P) = 176 Hz]; IR (solid) ν(CO): 1976 cm^-1^. IR (dichloromethane) ν(CO): 1981 cm^-1^

### Refinement

Hydrogen atom positions were calculated and refined using a riding model (C—H = 0.95–0.98 Å) with *U*_iso_(H) = 1.2*U*_eq_(C) for aromatic H atoms, and a riding model allowing the torsion angle to be refined from the electron density with *U*_iso_(H) = 1.5*U*_eq_(C) for methyl H atoms.

### Crystal data

**Table:**

[Rh(C_5_H_7_O_2_)(C_18_H_12_Cl_3_P)(CO)]	*Z* = 2
*M**_r_* = 595.62	*F*(000) = 596
Triclinic, *P*1	*D*_x_ = 1.628 Mg m^−^^3^
Hall symbol: -P 1	Mo *K*α radiation, λ = 0.71073 Å
*a* = 9.6528 (17) Å	Cell parameters from 8738 reflections
*b* = 11.535 (2) Å	θ = 2.7–28.4°
*c* = 12.875 (2) Å	µ = 1.12 mm^−^^1^
α = 65.211 (3)°	*T* = 100 K
β = 72.095 (4)°	Block, yellow
γ = 72.757 (4)°	0.18 × 0.13 × 0.12 mm
*V* = 1214.9 (4) Å^3^	

### Data collection

**Table:**

Bruker APEX DUO 4K CCD diffractometer	6083 independent reflections
Radiation source: sealed tube	5395 reflections with *I* > 2σ(*I*)
Graphite monochromator	*R*_int_ = 0.029
Detector resolution: 8.4 pixels mm^-1^	θ_max_ = 28.7°, θ_min_ = 1.8°
φ and ω scans	*h* = −11→13
Absorption correction: multi-scan (*SADABS*; Bruker, 2008)	*k* = −15→15
*T*_min_ = 0.826, *T*_max_ = 0.877	*l* = −17→17
19382 measured reflections	

### Refinement

**Table:**

Refinement on *F*^2^	Primary atom site location: structure-invariant direct methods
Least-squares matrix: full	Secondary atom site location: difference Fourier map
*R*[*F*^2^ > 2σ(*F*^2^)] = 0.027	Hydrogen site location: inferred from neighbouring sites
*wR*(*F*^2^) = 0.063	H-atom parameters constrained
*S* = 1.01	*w* = 1/[σ^2^(*F*_o_^2^) + (0.0242*P*)^2^ + 0.9592*P*] where *P* = (*F*_o_^2^ + 2*F*_c_^2^)/3
6083 reflections	(Δ/σ)_max_ = 0.005
291 parameters	Δρ_max_ = 0.50 e Å^−^^3^
0 restraints	Δρ_min_ = −0.48 e Å^−^^3^

### Special details

**Table:**

Geometry. All e.s.d.'s (except the e.s.d. in the dihedral angle between two l.s. planes) are estimated using the full covariance matrix. The cell e.s.d.'s are taken into account individually in the estimation of e.s.d.'s in distances, angles and torsion angles; correlations between e.s.d.'s in cell parameters are only used when they are defined by crystal symmetry. An approximate (isotropic) treatment of cell e.s.d.'s is used for estimating e.s.d.'s involving l.s. planes.
Refinement. Refinement of *F*^2^ against ALL reflections. The weighted *R*-factor *wR* and goodness of fit *S* are based on *F*^2^, conventional *R*-factors *R* are based on *F*, with *F* set to zero for negative *F*^2^. The threshold expression of *F*^2^ > σ(*F*^2^) is used only for calculating *R*-factors(gt) *etc*. and is not relevant to the choice of reflections for refinement. *R*-factors based on *F*^2^ are statistically about twice as large as those based on *F*, and *R*- factors based on ALL data will be even larger.

### Fractional atomic coordinates and isotropic or equivalent isotropic displacement parameters (Å^2^)

**Table:**

	*x*	*y*	*z*	*U*_iso_*/*U*_eq_	
Rh1	1.052699 (17)	0.359470 (15)	0.333583 (13)	0.01682 (5)	
Cl1	0.39474 (6)	0.72923 (6)	0.04828 (5)	0.03653 (14)	
Cl2	1.24276 (7)	0.04921 (6)	−0.10196 (5)	0.03470 (14)	
Cl3	0.52995 (6)	−0.10417 (5)	0.72212 (5)	0.02947 (12)	
P1	0.89407 (5)	0.28944 (5)	0.28971 (4)	0.01573 (10)	
O1	0.96017 (16)	0.28916 (14)	0.50713 (12)	0.0221 (3)	
O2	1.20480 (15)	0.42067 (14)	0.37193 (13)	0.0230 (3)	
O3	1.18810 (19)	0.46211 (18)	0.08083 (14)	0.0365 (4)	
C1	1.0041 (2)	0.2905 (2)	0.59045 (19)	0.0245 (4)	
C2	0.9110 (3)	0.2347 (3)	0.7102 (2)	0.0373 (6)	
H2A	0.863	0.3036	0.7426	0.056*	
H2B	0.9742	0.1658	0.7615	0.056*	
H2C	0.8349	0.1984	0.7045	0.056*	
C3	1.1259 (2)	0.3395 (2)	0.57878 (19)	0.0259 (5)	
H3	1.1492	0.3296	0.649	0.031*	
C4	1.2168 (2)	0.4018 (2)	0.4741 (2)	0.0235 (4)	
C5	1.3406 (3)	0.4541 (2)	0.4762 (2)	0.0325 (5)	
H5A	1.4362	0.4059	0.4468	0.049*	
H5B	1.3355	0.4441	0.5567	0.049*	
H5C	1.331	0.5465	0.4265	0.049*	
C6	0.7434 (2)	0.41545 (19)	0.22884 (17)	0.0184 (4)	
C7	0.7514 (2)	0.5465 (2)	0.18792 (18)	0.0229 (4)	
H7	0.8327	0.57	0.1962	0.027*	
C8	0.6419 (3)	0.6428 (2)	0.13528 (19)	0.0275 (5)	
H8	0.647	0.732	0.1086	0.033*	
C9	0.5257 (2)	0.6074 (2)	0.12224 (18)	0.0247 (4)	
C10	0.5121 (2)	0.4789 (2)	0.1645 (2)	0.0275 (5)	
H10	0.4301	0.4563	0.1563	0.033*	
C11	0.6208 (2)	0.3830 (2)	0.2192 (2)	0.0247 (4)	
H11	0.6115	0.2944	0.2505	0.03*	
C12	0.9830 (2)	0.21278 (19)	0.18053 (17)	0.0175 (4)	
C13	1.1223 (2)	0.1333 (2)	0.18776 (19)	0.0266 (5)	
H13	1.1643	0.1155	0.2518	0.032*	
C14	1.2014 (3)	0.0792 (2)	0.10336 (19)	0.0278 (5)	
H14	1.2958	0.0239	0.1099	0.033*	
C15	1.1402 (2)	0.1073 (2)	0.00991 (18)	0.0240 (4)	
C16	1.0022 (3)	0.1842 (3)	0.0012 (2)	0.0335 (5)	
H16	0.9606	0.2013	−0.0629	0.04*	
C17	0.9235 (2)	0.2369 (2)	0.0866 (2)	0.0285 (5)	
H17	0.8279	0.2901	0.0805	0.034*	
C18	0.7931 (2)	0.17036 (19)	0.41102 (17)	0.0177 (4)	
C19	0.8076 (2)	0.0432 (2)	0.41969 (18)	0.0224 (4)	
H19	0.8742	0.014	0.3599	0.027*	
C20	0.7263 (2)	−0.0419 (2)	0.51444 (19)	0.0246 (4)	
H20	0.7369	−0.1286	0.5197	0.03*	
C21	0.6299 (2)	0.0015 (2)	0.60070 (18)	0.0206 (4)	
C22	0.6129 (2)	0.1278 (2)	0.59502 (19)	0.0257 (5)	
H22	0.5459	0.1565	0.6549	0.031*	
C23	0.6956 (2)	0.2108 (2)	0.50032 (19)	0.0245 (4)	
H23	0.6859	0.297	0.496	0.029*	
C24	1.1342 (2)	0.4217 (2)	0.17864 (19)	0.0232 (4)	

### Atomic displacement parameters (Å^2^)

**Table:**

	*U*^11^	*U*^22^	*U*^33^	*U*^12^	*U*^13^	*U*^23^
Rh1	0.01580 (8)	0.02264 (8)	0.01419 (8)	−0.00561 (6)	−0.00310 (6)	−0.00747 (6)
Cl1	0.0251 (3)	0.0402 (3)	0.0282 (3)	0.0055 (2)	−0.0093 (2)	−0.0025 (2)
Cl2	0.0464 (3)	0.0316 (3)	0.0238 (3)	−0.0026 (3)	−0.0002 (2)	−0.0161 (2)
Cl3	0.0293 (3)	0.0268 (3)	0.0266 (3)	−0.0137 (2)	0.0029 (2)	−0.0049 (2)
P1	0.0154 (2)	0.0184 (2)	0.0137 (2)	−0.00436 (19)	−0.00333 (18)	−0.00522 (19)
O1	0.0227 (7)	0.0290 (8)	0.0157 (7)	−0.0058 (6)	−0.0035 (6)	−0.0088 (6)
O2	0.0185 (7)	0.0307 (8)	0.0263 (8)	−0.0054 (6)	−0.0052 (6)	−0.0156 (7)
O3	0.0359 (9)	0.0545 (11)	0.0200 (9)	−0.0231 (8)	0.0014 (7)	−0.0096 (8)
C1	0.0264 (11)	0.0272 (11)	0.0195 (10)	0.0001 (9)	−0.0068 (9)	−0.0101 (9)
C2	0.0447 (15)	0.0505 (15)	0.0177 (11)	−0.0151 (12)	−0.0054 (10)	−0.0099 (11)
C3	0.0277 (11)	0.0337 (12)	0.0236 (11)	0.0010 (9)	−0.0128 (9)	−0.0167 (9)
C4	0.0204 (10)	0.0243 (10)	0.0328 (12)	0.0041 (8)	−0.0119 (9)	−0.0183 (9)
C5	0.0254 (12)	0.0406 (13)	0.0466 (15)	−0.0015 (10)	−0.0138 (11)	−0.0289 (12)
C6	0.0170 (9)	0.0226 (9)	0.0160 (9)	−0.0037 (8)	−0.0040 (7)	−0.0072 (8)
C7	0.0238 (10)	0.0229 (10)	0.0236 (11)	−0.0087 (8)	−0.0066 (8)	−0.0060 (9)
C8	0.0304 (12)	0.0213 (10)	0.0242 (11)	−0.0043 (9)	−0.0065 (9)	−0.0020 (9)
C9	0.0188 (10)	0.0299 (11)	0.0166 (10)	0.0012 (8)	−0.0039 (8)	−0.0040 (9)
C10	0.0164 (10)	0.0349 (12)	0.0303 (12)	−0.0069 (9)	−0.0065 (9)	−0.0085 (10)
C11	0.0193 (10)	0.0240 (10)	0.0298 (12)	−0.0069 (8)	−0.0061 (9)	−0.0063 (9)
C12	0.0186 (9)	0.0198 (9)	0.0145 (9)	−0.0073 (7)	−0.0014 (7)	−0.0055 (8)
C13	0.0288 (11)	0.0299 (11)	0.0198 (11)	−0.0006 (9)	−0.0087 (9)	−0.0086 (9)
C14	0.0277 (11)	0.0267 (11)	0.0229 (11)	0.0017 (9)	−0.0043 (9)	−0.0088 (9)
C15	0.0315 (11)	0.0218 (10)	0.0176 (10)	−0.0075 (9)	0.0017 (9)	−0.0092 (8)
C16	0.0346 (13)	0.0486 (14)	0.0254 (12)	−0.0020 (11)	−0.0114 (10)	−0.0215 (11)
C17	0.0232 (11)	0.0396 (13)	0.0289 (12)	0.0001 (9)	−0.0092 (9)	−0.0199 (10)
C18	0.0170 (9)	0.0204 (9)	0.0156 (9)	−0.0055 (7)	−0.0053 (7)	−0.0039 (8)
C19	0.0239 (10)	0.0224 (10)	0.0204 (10)	−0.0037 (8)	−0.0026 (8)	−0.0093 (8)
C20	0.0283 (11)	0.0185 (9)	0.0255 (11)	−0.0060 (8)	−0.0034 (9)	−0.0071 (9)
C21	0.0183 (10)	0.0228 (10)	0.0190 (10)	−0.0075 (8)	−0.0040 (8)	−0.0035 (8)
C22	0.0241 (11)	0.0271 (11)	0.0243 (11)	−0.0074 (9)	0.0031 (9)	−0.0119 (9)
C23	0.0264 (11)	0.0216 (10)	0.0257 (11)	−0.0072 (8)	−0.0006 (9)	−0.0104 (9)
C24	0.0204 (10)	0.0307 (11)	0.0235 (11)	−0.0096 (9)	−0.0053 (8)	−0.0110 (9)

### Geometric parameters (Å, º)

**Table:**

Rh1—C24	1.812 (2)	C7—H7	0.95
Rh1—O1	2.0324 (14)	C8—C9	1.378 (3)
Rh1—O2	2.0616 (14)	C8—H8	0.95
Rh1—P1	2.2280 (6)	C9—C10	1.381 (3)
Cl1—C9	1.745 (2)	C10—C11	1.390 (3)
Cl2—C15	1.744 (2)	C10—H10	0.95
Cl3—C21	1.741 (2)	C11—H11	0.95
P1—C6	1.826 (2)	C12—C17	1.386 (3)
P1—C18	1.826 (2)	C12—C13	1.390 (3)
P1—C12	1.829 (2)	C13—C14	1.389 (3)
O1—C1	1.276 (2)	C13—H13	0.95
O2—C4	1.277 (2)	C14—C15	1.378 (3)
O3—C24	1.150 (3)	C14—H14	0.95
C1—C3	1.392 (3)	C15—C16	1.372 (3)
C1—C2	1.502 (3)	C16—C17	1.388 (3)
C2—H2A	0.98	C16—H16	0.95
C2—H2B	0.98	C17—H17	0.95
C2—H2C	0.98	C18—C19	1.391 (3)
C3—C4	1.391 (3)	C18—C23	1.396 (3)
C3—H3	0.95	C19—C20	1.390 (3)
C4—C5	1.502 (3)	C19—H19	0.95
C5—H5A	0.98	C20—C21	1.380 (3)
C5—H5B	0.98	C20—H20	0.95
C5—H5C	0.98	C21—C22	1.390 (3)
C6—C7	1.394 (3)	C22—C23	1.385 (3)
C6—C11	1.395 (3)	C22—H22	0.95
C7—C8	1.387 (3)	C23—H23	0.95
			
C24—Rh1—O1	179.59 (8)	C8—C9—Cl1	118.52 (17)
C24—Rh1—O2	91.04 (8)	C10—C9—Cl1	119.74 (17)
O1—Rh1—O2	89.37 (6)	C9—C10—C11	118.7 (2)
C24—Rh1—P1	88.08 (7)	C9—C10—H10	120.6
O1—Rh1—P1	91.50 (4)	C11—C10—H10	120.6
O2—Rh1—P1	178.23 (4)	C10—C11—C6	120.8 (2)
C6—P1—C18	101.96 (9)	C10—C11—H11	119.6
C6—P1—C12	104.31 (9)	C6—C11—H11	119.6
C18—P1—C12	104.69 (9)	C17—C12—C13	118.31 (18)
C6—P1—Rh1	115.10 (7)	C17—C12—P1	123.42 (15)
C18—P1—Rh1	116.37 (6)	C13—C12—P1	118.10 (15)
C12—P1—Rh1	112.95 (7)	C14—C13—C12	121.5 (2)
C1—O1—Rh1	126.58 (14)	C14—C13—H13	119.3
C4—O2—Rh1	126.09 (14)	C12—C13—H13	119.3
O1—C1—C3	126.0 (2)	C15—C14—C13	118.7 (2)
O1—C1—C2	114.7 (2)	C15—C14—H14	120.6
C3—C1—C2	119.3 (2)	C13—C14—H14	120.6
C1—C2—H2A	109.5	C16—C15—C14	121.06 (19)
C1—C2—H2B	109.5	C16—C15—Cl2	119.43 (17)
H2A—C2—H2B	109.5	C14—C15—Cl2	119.46 (17)
C1—C2—H2C	109.5	C15—C16—C17	119.8 (2)
H2A—C2—H2C	109.5	C15—C16—H16	120.1
H2B—C2—H2C	109.5	C17—C16—H16	120.1
C4—C3—C1	126.21 (19)	C12—C17—C16	120.7 (2)
C4—C3—H3	116.9	C12—C17—H17	119.7
C1—C3—H3	116.9	C16—C17—H17	119.7
O2—C4—C3	125.59 (19)	C19—C18—C23	118.53 (19)
O2—C4—C5	114.7 (2)	C19—C18—P1	124.22 (15)
C3—C4—C5	119.7 (2)	C23—C18—P1	117.25 (15)
C4—C5—H5A	109.5	C20—C19—C18	120.98 (19)
C4—C5—H5B	109.5	C20—C19—H19	119.5
H5A—C5—H5B	109.5	C18—C19—H19	119.5
C4—C5—H5C	109.5	C21—C20—C19	119.05 (19)
H5A—C5—H5C	109.5	C21—C20—H20	120.5
H5B—C5—H5C	109.5	C19—C20—H20	120.5
C7—C6—C11	118.78 (18)	C20—C21—C22	121.51 (19)
C7—C6—P1	120.16 (15)	C20—C21—Cl3	120.00 (16)
C11—C6—P1	121.04 (15)	C22—C21—Cl3	118.47 (16)
C8—C7—C6	120.7 (2)	C23—C22—C21	118.60 (19)
C8—C7—H7	119.6	C23—C22—H22	120.7
C6—C7—H7	119.6	C21—C22—H22	120.7
C9—C8—C7	119.1 (2)	C22—C23—C18	121.32 (19)
C9—C8—H8	120.5	C22—C23—H23	119.3
C7—C8—H8	120.5	C18—C23—H23	119.3
C8—C9—C10	121.73 (19)	O3—C24—Rh1	178.57 (19)
